# Laminin-5, Fibronectin, and Type IV Collagen as Potential Biomarkers of Brain Glioma Malignancy

**DOI:** 10.3390/biomedicines10092290

**Published:** 2022-09-15

**Authors:** Lukasz Oldak, Sylwia Chludzinska-Kasperuk, Patrycja Milewska, Kamil Grubczak, Joanna Reszec, Ewa Gorodkiewicz

**Affiliations:** 1 Bioanalysis Laboratory, Faculty of Chemistry, University of Bialystok, Ciolkowskiego 1K, 15-245 Bialystok, Poland; 2 Doctoral School of Exact and Natural Science, Faculty of Chemistry, University of Bialystok, Ciolkowskiego 1K, 15-245 Bialystok, Poland; 3 Biobank, Medical University of Bialystok, Waszyngtona 13, 15-269 Bialystok, Poland; 4 Department of Regenerative Medicine and Immune Regulation, Medical University of Bialystok, Waszyngtona 13, 15-269 Bialystok, Poland; 5 Department of Medical Pathology, Medical University of Bialystok, Waszyngtona 13, 15-269 Bialystok, Poland

**Keywords:** glioma, laminin-5, fibronectin, type IV collagen, SPRi biosensors, basement membrane in carcinoma

## Abstract

The presented work is based on the quantification of LN-5, FN, and COL IV in blood plasma as potential biomarkers in patients diagnosed with glioma in grades G1 to G4. The obtained concentration results were compared with the protein content in the control group, which consisted of smokers of different ages. The obtained results were statistically analysed and interpreted based on the available clinical description. Quantitative determinations of LN-5, FN, and COL IV were performed with the use of SPRi biosensors specific to the tested proteins. Comparing groups K and G4, as well as G2 and G4, statistically significant relationships between changes in the concentration of individual proteins, were observed. The analysis showed significant correlations between FN and LN-5, between FN and COL IV, and between LN-5 and COL IV. There was a moderate positive correlation between individual proteins and the age of the patient. The ROC analysis distinguished patients with advanced disease from the control group. The results of the research show that LN-5, FN, and COL IV are effective biomarkers of brain glioma that may be helpful in non-invasive diagnosis and determining the grade of the disease.

## 1. Introduction

The basement membrane (BM) is a structure that consists of extracellular matrix (ECM) proteins. The main structural proteins of the BM are laminin-5 (LN-5) and type IV collagen (COL-IV). Fibronectin (FN), although located in close proximity to the BM, is not one of the strategic structural proteins of the BM. The BM functions primarily as a mechanical barrier. In addition, it influences cell proliferation, adhesion, differentiation, and migration. BM molecules are often responsible for regulating the activity of growth factors, which in turn participate in the regulation of epithelial cell differentiation and development, leading to further processes [1]. Normal and mature nervous parenchyma contains its own characteristic ECM. It acts as a barrier to cell motility and neurites. Gliomas disrupt the ECM and then diffuse into the neural tissue, increasingly displacing it as the tumour grows [2].

Increased levels of LN-5 in glioma tumour cells correlate with their increased invasiveness [3]. LN-5 and its other isoforms have been localised primarily in the vascular system of brain tumours. Reports in the available literature indicate that it is the γ2 chain that is associated with the invasiveness of human malignant neoplasms. Neoplasms such as breast, lung, pancreatic, colon, and cervical cancer are mainly mentioned [1]. Liquid biopsy studies on LN-5 levels have not been previously conducted. The available data show immunohistochemical analyses of primary samples of brain glioma. An association was noted between increased LN-5 γ2 expression and invasiveness at all four grades [3]. The presence of LN-5 was also observed within the tumour itself in the form of punctate deposits, as well as in boundary areas between the tumour and healthy brain tissue. LN-5 promotes the migration of glioma cells strongly and efficiently [4]. This gives rise to the idea that LN-5 plays a dual role in tumour progression. In tumours such as glioma, colon cancer, and gastric tract cancer, LN-5 is a highly expressed marker for proliferating tumour cells located primarily at the invasive marginal region of the tumour. However, LN-5 behaves differently in basal cell carcinoma and prostate cancer, where its expression was shown to decrease relative to healthy tissues [5].

According to the available scientific literature, FN has two conflicting functions in oncogenesis and cancer progression. On the one hand, it is believed that FN is responsible for preventing tumour transformation and inhibiting tumour progression. On the other hand, other researchers argue that FN is responsible for late grades of cancer metastasis and, at the same time, for poor prognosis of the disease [6]. The presence of FN in glioma tumours and their surrounding spaces was confirmed by immunohistochemistry. In immunoblotting, the clear presence of FN was observed in the tumour tissues and in the adjacent spaces. Clear staining was seen in all malignant gliomas, a much lower intensity was observed in glioma and low-grade malignancy, and no staining was observed in healthy brain tissues [7,8]. Increased amounts of FN were observed primarily in samples of the tumour itself as well as in peripheral blood in patients mainly having glioblastoma multiforme (GBM) [9]. Previous studies already showed that FN might play an important role in tumour progression in GBM [10].

Type IV collagen belongs to the class of so-called cross-linking collagens. It forms structures called collagen sheets, making it the main component of the basement membrane. It is the main form of collagen in the brain. It is found in the basement membrane surrounding vascular endothelial cells. The amount of collagen IV in glioma increases quite significantly relative to a brain with no tumour. The greatest increases in collagen IV levels are observed in gliomas at all grades of the disease [11]. Collagen IV in glioma is responsible for the development of an extremely extensive network of microvessels. This is linked to the function that type IV collagen performs in key steps of tumour progression, such as cell adhesion, motility, and angiogenesis. Compared with healthy nerve cells, glioma cells have the ability to independently secrete proteins in the brain that are not expressed in the healthy ECM of the nerve parenchyma. These include the previously reported LN-5, FN, and type IV collagen [12,13,14]. As a result, COL IV is thought to be one of the key ECM proteins responsible for growth and tumour invasion. This finding applies mainly to solid and diffuse tumours, primarily in GBM. It is likely that the network structure formed by COL IV is one of the main factors in the progression of glioma tumours, with a low degree of malignancy towards tumours of increasing malignancy, leading to increased disease progression [15].

FN and LN-5 are among the major ECM proteins. FN genes are among the most upregulated genes in brain glioma compared to normal brain tissue. The overall increase in laminins allows for the differentiation of the tumour class [16]. COL IV levels are generally elevated in all grades of gliomas, although there is virtually no collagen in the brain tissue, which makes the brain a soft structure. However, it was reported that GBM does not express fibrous collagen inside the tumour, but glioblastoma cells can synthesise their own ECM components, including collagen I and IV. In addition, growth factors, paracrine ligands, stromal cells, and the parenchyma of the unchanged brain surrounding the tumour contribute to the presence of ECM components in the tumour microenvironment and the production of COL IV [11].

Surface plasmon resonance (SPR) is a phenomenon that occurs in the surface layers of metals, most often gold or silver. It occurs when a beam of monochromatic and p-polarized light strikes the metal surface at a certain angle. Depending on the thickness of the layer on the metal surface (such as our sensor), the reflected light is reduced. This light then reaches the detector, which converts the light intensity into an analytical signal. The great advantage of the SPR technique is the lack of a need to use markers and the possibility of observing ligand–protein interactions in real time [17,18]. By using biosensors with SPR detection, the quantification of various potential biomarkers in various biological materials was successfully carried out, including quantification of plasma adalimumab [19], UCH-L1 in the serum [20], FN in the serum [21], and human cardiac troponin T in the serum [22]. The SPR was also used to study the activity of MMP-3 [23] and MMP-9 [24], as well as to determine the binding constant and the stoichiometry of the reaction between the antibody and antigen [25].

Brain tumour cells may interact with the ECM. This is a critical factor in the process of cancer invasion. The interaction takes place thanks to specific receptors located on the surface of the tumour cells. The main role in the adhesion of glioma cells to the ECM is played by integrins capable of binding the perivascular ECM, which includes collagen, fibronectin, and laminin. It was shown that in GBM tissues, as compared to healthy brains, integrins are highly expressed [13]; therefore, we suppose that LN-5, FN, and COL IV by binding to integrins may play an important role in tumour progression.

The present article presents the first data on concentration levels of LN-5 (full-length protein), FN, and COL IV in the blood plasma of people with brain glioma in grades G1 to G4, which are compared with concentrations of the tested proteins in the control group K. Appropriate statistical analysis was performed to determine the significance of the variation in concentration of the tested proteins between the grades, correlating the concentrations of LN-5, FN, and COL IV with data available in the clinical description of the studies, and determining how different potential biomarkers correlate with each other.

## 2. Materials and Methods

### 2.1. Reagents

The biosensor bases, which were gold plates with a sputtered gold thickness of 50 nm, were purchased from SSeens (Enschede, The Netherlands). They were then suitably prepared for use in quantification by printing the relevant polymers, following the procedure outlined in an earlier article [17]. Cysteamine hydrochloride and N-ethyl-N′-(3-dimethylaminopropyl)carbodiimide (EDC) were obtained from Sigma Steinheim, while N-hydroxysuccinimide (NHS), Tween-20, and N-(2-hydroxyethyl)piperazine-N′-(2-ethanesulforicacid) (HEPES) were purchased from Aldrich (Saint Louis, MO, USA). POCh Gliwice supplied absolute ethanol, sodium hydroxide, sodium chloride, and sodium carbonate. The PBS buffer (phosphate buffered saline, pH = 7.4) was from Biomed (Lublin, Poland). The following buffers were also used during the study: carbonate buffer pH = 8.50, HBS-ES pH = 7.40 (0.01 M HEPES, 0.15 M sodium chloride, 0.005% Tween-20, 3 mM EDTA). Recombinant human laminin-5 (ab42326, full-length protein) and rabbit polyclonal antibody specific to human laminin-5 (ab14509) were purchased from Abcam (Cambridge, UK). Type IV collagen (CC076) was from Sigma (Saint Louis, MO, USA), and monoclonal mouse anti-human collagen type IV (CL60411AP-1) was obtained from Tebu-bio (Le Perray-en-Yvelines Cedex, France). Sigma (Saint Louis, MO, USA) also supplied fibronectin from human plasma (F4759, as standard, lyophilised powder) and rabbit monoclonal anti-fibronectin antibody (F3648).

### 2.2. Biological Material

During the study, 104 plasma samples were available (48 control samples, 3 samples from grade G1, 10 samples from G2, 7 samples from G3, and 36 samples from G4). Biological material was supplied by the Biobank of the Medical University of Bialystok. The study was approved by the appropriate bioethics committee (permission APK.002.171.2021). Table 1 shows the clinical characteristics of the patients from whom the biological material was obtained.

### 2.3. Procedure for Quantifying LN-5, FN, COL IV

Previously constructed SPRi biosensors sensitive to individual proteins were used for quantification. On each occasion, the developed analytical procedure was reproduced according to the potential biomarker under study. An extremely important step is the appropriate and correct calibration of each analytical method; this process was therefore repeated before the quantification of subsequent proteins. Details of the analytical procedures were presented in earlier articles (references in Table 2). Each of the methods used was previously validated and presented in detail in previous works (references in Table 2). Nevertheless, for the purposes of the research presented in this article, each of them underwent slight modifications. They are concerned with the calibration process and sample dilution. The calibration of the analytical methods was performed each time before the quantitative determinations, and the calibration procedure was described in Section 2.4 SPRi measurements, while the fold of the dilution was selected experimentally so that the analytical signal fell within the rectilinear range of the calibration curve. Apart from centrifugation of the blood and appropriate dilution of the obtained plasma with PBS buffer, the samples did not require a more extensive preparation procedure for testing. Table 2 lists the most important characteristics of the procedure and the necessary elements of the procedure for the quantification of LN-5, FN, and COL IV in the glioma and control groups.

### 2.4. SPRi Measurements

**Figure 1:**
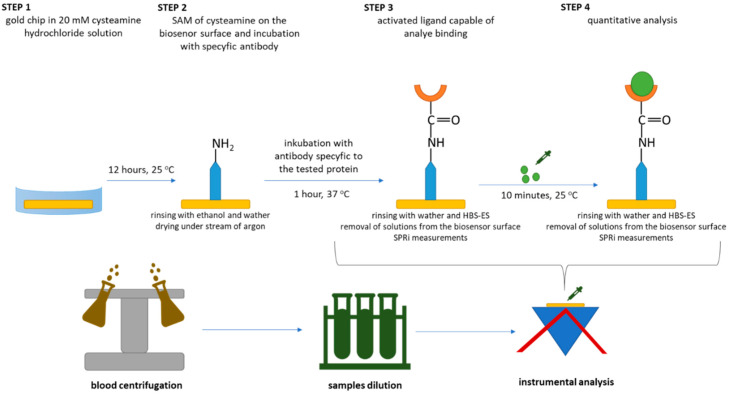
Procedure for quantitative determination of tested proteins.

Quantification of LN-5, FN, and COL IV was carried out using biosensors in combination with the SPR imaging (SPRi) technique, which was used as the detection method. The device used is characterised by an optical system following the classical Kretschmann configuration [28]. The SPRi spectrometer consists of, among other components, a light source, which is a laser diode (λ = 635 nm), lenses focusing the radiation beam, and polarisers responsible for selecting the appropriate polarisation. The beam of monochromatic p- or s-polarised radiation is then reflected from the metallic layer of the biosensor and falls onto the lens of a 1.4 MP CCD camera, which acts as a detector. SPRi measurements were carried out in two polarisations. The p-polarisation was used to observe changes in the intensity of active sites of the biosensor after binding successive layers of the biosensor, whereas the s-polarisation was the source of images of interference coming from the optical system of the device. It is important to take this into account and correct for changes in the intensity of the beam of radiation, exciting plasmons depending on the position of the arm on which the laser diode is placed. A detailed description and schematic illustration of the SPRi device used during the experiments were presented in earlier works [17]. The analytical signals obtained were then read using ImageJ software (National Institutes of Health, NIH) to obtain values of the concentration (after taking dilutions into account) of the given protein in the studied sample. The quantification procedure is shown in Figure 1.

### 2.5. Methods Calibration and Quantitative Determinations

**Figure A1:**
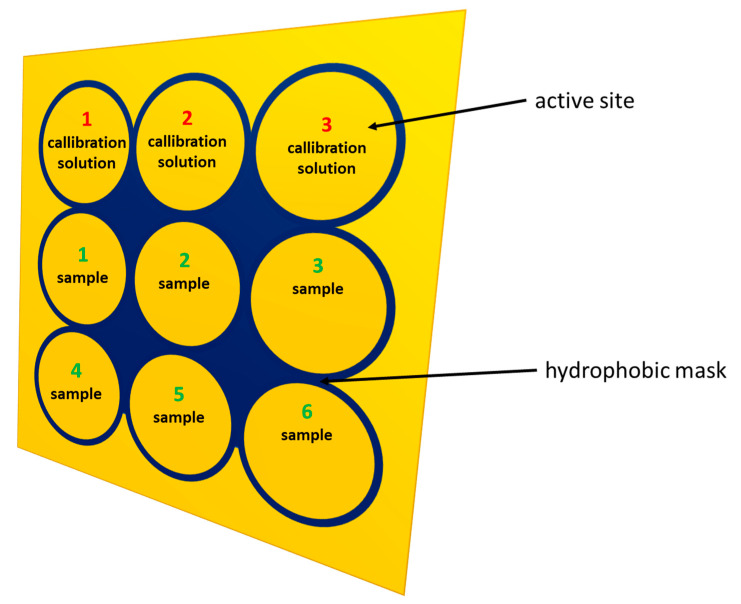
Scheme of biosensors used during the research.

Each analysis was begun by placing the base of the biosensor—gold leaf—in a 20 mM thiol (cysteamine) solution, which is the link between the gold and the ligand (antibody), for 12 h at 25 °C. The chip was then rinsed with ethanol and dried under a 99.98% pure argon stream. In the next step, the antibody solution, previously activated with EDC and NHS, was applied to the surface of the biosensor. The chip was placed in an incubator for 1 h at a constant temperature of 37 °C. After this time, the chip was rinsed with distilled water and HBS-ES diluent to remove nonspecifically bound ligands from the biosensor surface. A series of images were taken before the ligand–analyte interaction, and the optimal range of angles was selected. Photographs were taken in p- and s-polarisation. Calibration of analytical methods was performed each time before starting quantitative determinations of a given potential biomarker. For this purpose, three standard protein solutions were prepared, the concentrations of which included the first, middle, and last points from the rectilinear range of the previously drawn calibration curve in a wide range of concentrations. Three out of nine active biosensor sites were used for calibration. Figure A1 in Appendix A shows a schematic of the biosensors used in our research. Each time the obtained calibration curve was compared to its prototype in order to detect irregularities in the functioning of the biosensor. After the approval of the calibration process, the quantitative determinations were started. Appropriately diluted samples were applied to the surface of the biosensor and left at room temperature for 10 min until the ligand–protein interaction was complete. The surface of the biosensor was again washed with water and HBS-ES, and the excess of the solutions used was removed by suction. As before, a series of photographs were taken over a set range of angles. The data obtained were mathematically processed to obtain information on the quantitative content of LN-5, FN, or COL IV in the tested sample.

## 3. Results

### 3.1. Statistical Analysis

Statistical analysis was performed using the commercial software PQStat (PQStat Software (2022), PQStat v.1.8.4, Poznan, Poland).

**Figure 2:**
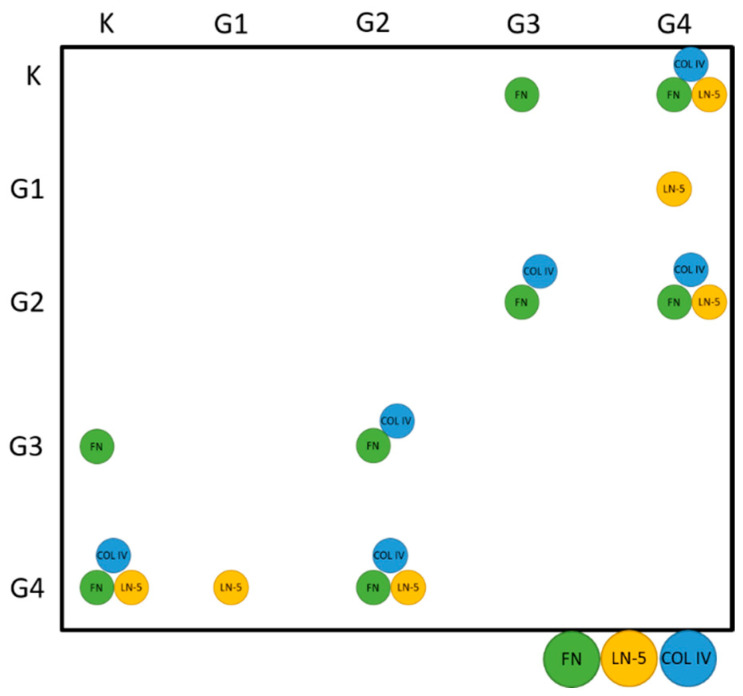
Graphical representation of the results of Dunn–Bonferroni analysis as a post hoc test.

In order to check whether the data followed a normal distribution, the Shapiro–Wilk test was performed under the assumption of H_0_, stating the presence of a normal distribution. Since the *p*-values satisfied *p* << α (where α = 0.05), it was assumed that the values of concentrations obtained during the study did not follow a normal distribution for any of the analysed proteins. Therefore, the analyses were performed using non-parametric tests. The first of these was the Kruskal–Wallis test, where H_0_ was taken as asserting the equality of medians in all tested groups (from K to G4), while H_1_ was the statement that not all medians were equal. In each case, *p* << α, and hence H_0_ was rejected in favour of H_1_. This entailed performing a Dunn–Bonferroni analysis as a post hoc test, the results of which are presented graphically in Figure 2.

The above data show that the concentrations of all of the proteins studied are statistically significantly different between K and G4 and between G2 and G4. Statistically significant changes in FN and COL IV concentrations were also observed between groups G2 and G3. The level of FN is also significantly increased in G3 compared with K, while the concentration of LN-5 is significantly increased in G4 compared with G1.

**Figure 3:**
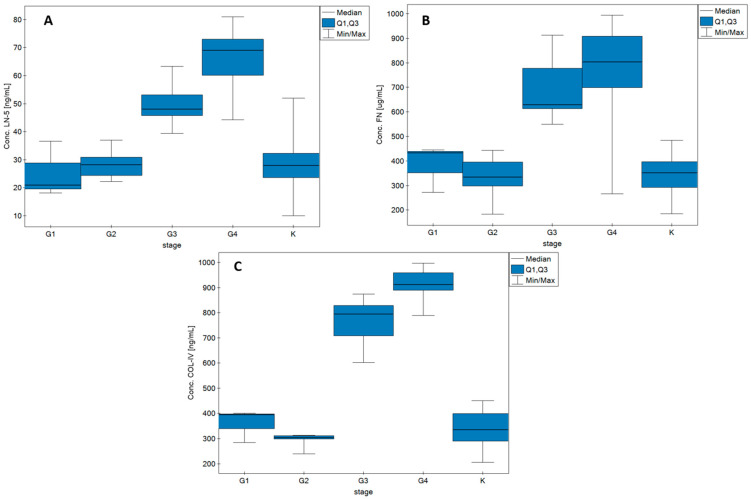
Box plots showing changes in concentrations of (**A**) LN-5, (**B**) FN, and (**C**) COL IV according to disease grade and in the control group.

Figure 3 shows the changes in LN-5, FN, and COL IV concentrations as functions of glioma grade. The graphs also show data on the concentrations of each protein in the control samples. Detailed data on median, Q1, and Q3 values and min/max ranges can be found in Table A1 in Appendix A.

Table 3 shows the characteristics of the changes in LN-5, FN, and COL IV concentrations depending on the grade of the disease and other data available in the clinical description of the samples: tumour size, presence of other cancers in the patient’s immediate family, and presence of comorbidities in the patient.

For the purposes of the statistical analysis, data on the median concentrations of individual proteins from the G1 and G2, as well as G3 and G4 grades, were combined. The table also shows the concentration range of individual proteins depending on the parameter tested (disease grade, tumour size, presence of other neoplastic diseases in the patient’s family, and the presence of non-neoplastic comorbidities in the patient). It can be seen from the table above that there are statistically significant differences in the concentrations of each of the proteins studied when considering G1 and G2 together compared with the combination of groups G3 and G4. No statistically significant differences were observed between the concentrations of LN-5, FN, and COL IV depending on tumour size in any of the cases studied. The same applies when considering the presence of other neoplastic diseases in the patient’s immediate family. The presence of other non-cancer comorbidities in the patient also does not affect the changes in concentrations of the tested potential biomarkers.

An attempt was also made to determine correlations between LN-5, FN, and COL IV and patient age and tumour size. Analysis of the data indicated the occurrence of positive average correlations (R_S_ = 0.42–0.49, *p* < 0.05) between concentrations of the examined proteins and patient age. This situation occurs only when the whole group of patients is analysed, without division into individual grades. In the case of correlation of concentrations of the analysed proteins with tumour area, no statistically significant correlations were observed, either after dividing the data into individual grades of the disease or on analysing all of the samples as a whole. All calculated numerical values are presented in Table A2 in Appendix A.

**Figure 4:**
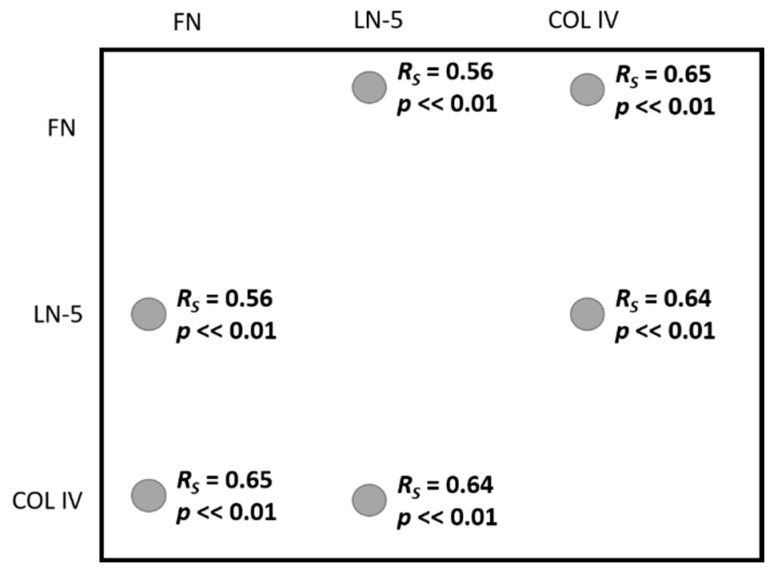
Correlation between LN-5, FN, and COL IV.

Figure 4 shows the interdependence between the proteins studied. As with the protein concentration–age correlation, the relationships are visible only when all samples are analysed simultaneously.

The Spearman correlation coefficient values R_S_ = 0.56–0.65 (*p* << 0.01) indicate a high positive correlation in pairs FN:LN-5, FN:COL IV, and LN-5:COL IV. The results of the full analysis can be found in Table A3 in Appendix A. Interpretation of the strength of correlation was based on J. Guilford’s scale.

### 3.2. ROC Analysis

**Figure 5:**
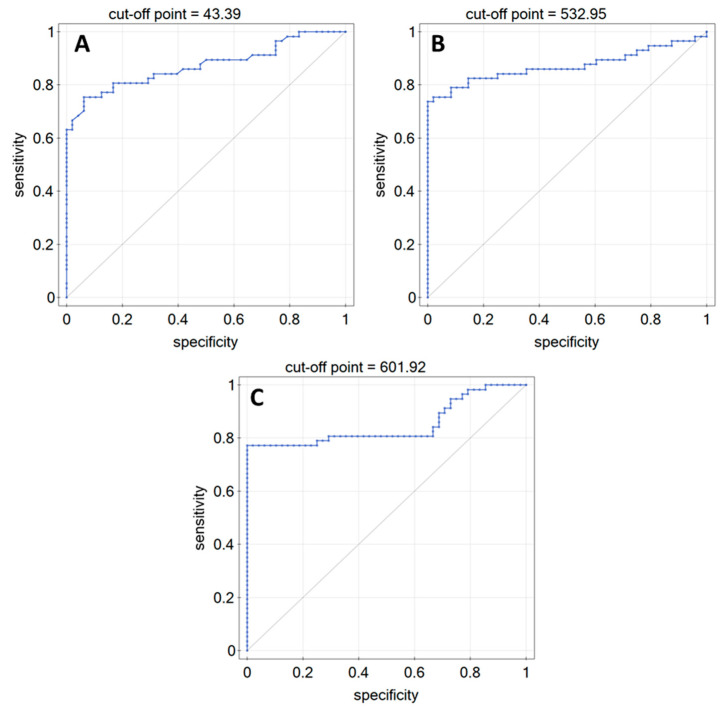
ROC curves: (**A**) LN-5, (**B**) FN, (**C**) COL IV.

In order to check whether, when examining changes in LN-5, FN, or COL IV concentrations, it was possible to distinguish patients diagnosed with glioma from the control group, ROC curves were produced, omitting the data from the G1 group due to the insufficient number of cases. Analysis of the curves showed that it is possible to distinguish patients with the disease from the control group, but only when taking into account the more advanced grades of the disease. The ROC curves are shown in Figure 5.

Table 4 shows data demonstrating the diagnostic efficacy of LN-5, FN, and COL IV in plasma from patients with brain glioma.

## 4. Discussion and Conclusions

Liquid biopsy is a promising and rapid diagnostic test to confirm or exclude the presence of disease. Newer, faster, cheaper, and less invasive analytical methods are being developed for the quantitative analysis of a variety of biological materials for screening or specialised studies. The work presented here is based on the quantitative determination of LN-5, FN, and COL IV in brain glioma, from which further observations of correlations were made, supported by the available clinical description of the samples studied.

The Kruskal–Wallis test showed that not all medians of the analysed groups were equal to each other, which was the first assumption after analysing the results of quantitative determinations. The obtained data were further clarified using Dunn–Bonferroni analysis as a post hoc test. This indicated which differences were statistically significant. It turned out that the concentrations of all studied proteins were statistically significantly different between the K and G4 groups and also between G2 and G4. When the differences in concentrations of the analysed proteins between milder grades of the disease (G1–G2) and more advanced grades (G3–G4) are analysed, statistically significant differences are observed in the concentrations of all of the analysed proteins (LN-5, FN, and COL IV) between the presented groups of patients.

The area of the neoplastic tumour, the presence of neoplastic diseases in the patient’s closest family, or the presence of coexisting diseases other than neoplasms in the patient did not affect the amount of LN-5, FN, or COL IV in plasma.

If we analyse the whole group of patients, i.e., all samples from grade G1 to G4, we observe an average positive correlation between the determined protein levels and the patient’s age, which means that the amounts of LN-5, FN, and COL IV in the body increase with age. However, it should be noted that samples at grade G4 constituted the majority of the samples analysed, and hence it is possible that this effect is due only to the size of the sample groups analysed. On the other hand, no correlation was found between the concentration of the analysed proteins and tumour size, as mentioned earlier.

A high positive correlation occurs between individual proteins only when all samples are analysed together. This positive correlation indicates that an increase in the concentration of one component entails an increase in the concentration of the other component. It is possible that such correlations may be used to develop an algorithm to diagnose glioma.

ROC curves were also plotted, the parameters of which indicate that control samples can be quite clearly distinguished from samples from subjects in grades G2–G4. The ROC curves indicate high or very high diagnostic efficacy of LN-5, FN, and COL IV in the plasma of patients with brain glioma.

With the tumour’s increase in malignancy, the levels of the individual proteins increase significantly; they remain similar in groups K–G2 and then increase, again maintaining similar levels in groups G3 and G4. We observed almost identical levels of LN-5, FN, and COL IV concentrations (considering each protein separately) in groups K, G1, and G2. This may be due to the fact that these proteins often have a dual function in tumours. On the one hand, they are responsible for inhibiting tumour’s increase in malignancy, while on the other, they contribute to tumour invasiveness [4,5,6,11]. It is noteworthy that glioma tumours can independently secrete certain proteins (including LN-5, FN, COL IV) [12,13,14], which can be expected to lead to a significant increase in their concentrations. As the studied proteins may, to some extent, have a suppressive function in the tumour process, the results suggest that the invasiveness of glioma increases dramatically in grades G3–G4.

The extracellular environment undergoes frequent changes during the pathogenesis of the disease. Changes in the composition of the ECM affect the functioning of the cells that make up the blood–brain barrier (BBB). These changes are carried out by the ECM receptors, which transmit appropriate signals to match the cell’s response to environmental changes. The expression of ECM proteins and the secretion of its proteins in the BBB are tightly regulated during the course of the disease development. Changes in BBB functioning were shown to correlate with changes in the composition of the ECM. The ECM proteins and their receptors, therefore, act as regulators of BBB functioning [29]. Since, as mentioned earlier, brain glioma tumours can synthesise some ECM proteins on their own, and the expression and secretion of ECM proteins in BBB are regulated during the disease, it is presumed that, in the G1/G2 BBB grades, it is not yet damaged enough to allow all proteins to cross it freely and access the bloodstream. It is likely that changes in the ECM composition of the diseased brain also have a similar effect on the functioning of the BBB.

The studied biomarkers can also function as biomarkers of other diseases. For example, all three biomarkers presented in the study (LN-5, FN, and COL IV) may be biomarkers of the epithelial-mesenchymal transition in squamous cell carcinoma [30]. In urothelial bladder cancer, LN-5 (more specifically, the γ2 chain) is recognised as an independent factor in TUR-treated bladder cancer. The altered distribution of the γ2 chain is associated with a worse prognosis for the patient and an increased risk of disease progression and recurrence [31,32,33,34]. Loss of COL IV in the basement membranes, on the other hand, is associated with increased tumour invasiveness and worse overall survival [34,35,36]. Increased FN expression is associated with increased expression of LN-5 and COL IV in urothelial bladder cancer. It also correlates with the grade of the disease, and its higher concentration in urine indicates the presence of early-grade cancer [37,38,39,40].

This study confirms that LN-5, FN, and COL IV are effective markers of brain glioma, despite the lack of correlation with tumour size and the impossibility of distinguishing the grade of the disease on the basis of LN-5, FN, or COL IV levels alone.

## Figures and Tables

**Table 1:** Clinicopathological characteristics of patients.

Variable	Range	Number of Patients
**Age [years]**	<50	16
	>50	40
**Gender**	male	34
	female	22
**Tumour grade**	G1	3
	G2	10
	G3	7
	G4	36
**Tumour size [cm^2^]**	<15	15
	>15	15
**The presence of other neoplasms in the immediate family**	yes	26
	no	30
**Concomitant non-cancerous diseases**	yes	28
	no	28

**Table 2:** Characteristics of analytical methods and procedures for quantifying the concentration of tested proteins.

Analytical Characteristics of the Methods Used				Characteristics of the Quantification Procedure Used		
Biomarker	Method	Analytical Characteristic	References	Biological Material	Dilution	pH
LN-5	SPRi biosensor [non fluidic]	C_antibody_ = 5 ng/mL LOD = 4 pg/mL LOQ = 14 pg/mL pH = 7.40 LR: 0.01–0.075 ng/mL	[18]	**Blood plasma** (blood centrifuged at 3000 rpm for 15 min then stored at −80 °C)	K G1 x1000 G2 G3 x1200 G4	7.40
FN	SPRi biosensor [non fluidic]	C_antibody_ = 4 µg/mL LOD = 1.5 ng/mL LOQ = 5 ng/mL pH = 7.40 LR: 5–100 µg/mL	[26]		K G1 x5 G2 G3 x10 G4	
COL IV	SPRi biosensor [non fluidic]	C_antibody_ = 6 µg/mL LOD = 2.4 ng/mL LOQ = 8 ng/mL pH = 7.40 LR: 10–150 ng/mL	[27]		K G1 x5 G2 G3 x10 G4	

**Table 3:** Changes in LN-5, FN, and COL IV concentrations in comparison with the available clinical data for the samples from patients.

Parameter	LN-5 *concentration* [ng/mL]			FN *concentration* [µg/mL]			COL IV *concentration* [ng/mL]		
	Range	Median	*p*-Value	Range	Median	*p*-Value	Range	Median	*p*-Value
Tumour grade (G1–G2 vs. G3–G4)									
G1 (3)	18.13–36.58	21.08	<0.01	271.82–444.88	432.36	<0.01	283.21–399.81	394.37	<0.01
G2 (10)	22.26–37.00	28.15		183.04–443.27	334.70		239.64–312.69	304.10	
G3 (7)	39.37–63.36	48.51		549.54–912.20	628.92		601.92–874.64	795.19	
G4 (36)	44.20–81.02	69.23		266.40–994.07	803.34		789.65–997.64	912.39	
Tumour size [cm^2^]									
<15 (15)	21.05–73.40	60.11	0.37 (NS)	329.36–994.07	651.40	0.74 (NS)	239.64–994.64	894.97	0.26 (NS)
>15 (15)	18.13–79.52	64.25		183.04–964.44	771.84		297.44–973.23	829.64	
The presence of other neoplasms in the family									
YES (26)	18.13–81.37	58.80	0.76 (NS)	266.40–994.07	747.56	0.77 (NS)	269.36–997.64	890.40	0.98 (NS)
NO (30)	22.26–81.42	61.04		183.04–978.62	651.40		239.64–973.23	879.48	
Concomitant non-cancerous diseases									
YES (28)	22.26–80.02	64.86	0.31 (NS)	271.82–946.79	763.37	0.43 (NS)	239.64–997.64	892.27	0.20 (NS)
NO (28)	18.13–81.42	59.71		183.04–994.07	719.45		269.36–994.64	871.97	

(NS)—no statistically significant values.

**Table 4:** Data on the diagnostic performance of the proteins tested.

	AUC	*p*-Value	PPV [%]	NPV [%]	Sensitivity [%]	Specificity [%]	Cut-Off Point
LN-5	0.87	<<0.01	93.5	76.3	75.4	93.8	43.39
FN	0.87	<<0.01	100	76.2	73.7	100	532.95
COL IV	0.85	<<0.01	100	78.7	77.2	100	601.92

## Data Availability

Not applicable.

## Appendix A

**Table A1:** Detailed data for Figure 3.

Paramete/ Biomarker	LN-5 [ng/mL]					FN [µg/mL]					COL IV [ng/mL]				
	K	G1	G2	G3	G4	K	G1	G2	G3	G4	K	G1	G2	G3	G4
**Median**	27.89	21.52	28.15	48.04	69.75	351.41	432.36	334.71	628.92	803.34	334.35	394.37	304.09	795.19	912.39
**Q1**	32.25	28.79	30.91	53.19	73.00	396.47	438.62	395.04	777.51	908.45	398.82	397.09	310.09	829.04	958.91
**Q3**	23.57	19.57	24.32	45.70	60.11	292.16	352.09	297.42	613.45	698.63	288.69	338.79	297.35	708.64	889.69
**MIN**	10.04	18.10	22.20	39.37	44.20	184.14	271.82	183.04	549.54	266.40	205.61	283.21	239.64	601.92	789.65
**MAX**	52.23	36.58	37.00	63.36	81.00	484.18	444.98	443.27	912.20	994.07	449.87	399.81	312.69	874.64	997.64

**Table A2:** Correlations: protein concentration—patient’s age and protein concentration—tumour size.

Age—FN					
	**G1**	**G2**	**G3**	**G4**	**All samples**
**R_Spearman_**	−1	0.29	−0.27	0.02	0.42
**p**	-	>0.05	>0.05	>0.05	<0.05
**Age—LN-5**					
	**G1**	**G2**	**G3**	**G4**	**All samples**
**R_Spearman_**	1	−0.13	−0.17	0.01	0.49
**p**	-	>0.05	>0.05	>0.05	<0.05
**Age—COL IV**					
	**G1**	**G2**	**G3**	**G4**	**All samples**
**R_Spearman_**	−0.5	−0.48	0.45	−0.05	0.47
**p**	>0.05	>0.05	>0.05	>0.05	<0.05
**Tumour size—FN**					
	**G1**	**G2**	**G3**	**G4**	**All samples**
**R_Spearman_**	-	−0.6	0	−0.04	−0.01
**p**	-	>0.05	>0.05	>0.05	>0.05
**Tumour size—LN-5**					
	**G1**	**G2**	**G3**	**G4**	**All samples**
**R_Spearman_**	-	−0.4	−0.2	0.2	0.16
**p**	-	>0.05	>0.05	>0.35	>0.05
**Tumour size–COL IV**					
	**G1**	**G2**	**G3**	**G4**	**All samples**
**R_Spearman_**	-	−0.4	0.8	−0.46	−0.19
**p**	-	>0.05	>0.05	<0.05	>0.05

**Table A3:** Correlation analysis between proteins.

G1			
	**FN—LN5**	**FN—COL4**	**LN-5 COL4**
**R_Spearman_**	−1	0.5	−0.5
**p**	>0.05	>0.05	>0.05
**G2**			
	**FN—LN5**	**FN—COL4**	**LN-5 COL4**
**R_Spearman_**	−0.16	−0.37	0.018
**p**	>0.05	>0.05	>0.05
**G3**			
	**FN—LN5**	**FN—COL4**	**LN-5 COL4**
**R_Spearman_**	−0.50	0.57	−0.72
**p**	>0.05	>0.05	>0.05
**G4**			
	**FN—LN5**	**FN—COL4**	**LN-5 COL4**
**R_Spearman_**	0.03	0.16	−0.02
**p**	>0.05	>0.05	>0.05
**All samples**			
	**FN—LN5**	**FN—COL4**	**LN-5 COL4**
**R_Spearman_**	0.56	0.65	0.64
**p**	<<0.01	<<0.01	<<0.01

## References

[1] Lohi J. (2001). Laminin-5 in the Progression of Carcinomas. Int. J. Cancer.

[2] Hu B., Kong L.L., Matthews R.T., Viapiano M.S. (2008). The Proteoglycan Brevican Binds to Fibronectin after Proteolytic Cleavage and Promotes Glioma Cell Motility. J. Biol. Chem..

[3] Quo P., Imanishi Y., Cackowski F.C., Jarzynka M.J., Tao H.Q., Nishikawa R., Hirose T., Hu B., Cheng S.Y. (2005). Up-Regulation of Angiopoietin-2, Matrix Metalloprotease-2, Membrane Type 1 Metalloprotease, and Laminin 5 γ 2 Correlates with the Invasiveness of Human Glioma. Am. J. Pathol..

[4] Kawataki T., Yamane T., Naganuma H., Rousselle P., Andurén I., Tryggvason K., Patarroyo M. (2007). Laminin Isoforms and Their Integrin Receptors in Glioma Cell Migration and Invasiveness: Evidence for a Role of A5-Laminin(s) and A3β1 Integrin. Exp. Cell Res..

[5] Martin K.J., Kwan C.P., Nagasaki K., Zhang X., O’Hare M.J., Kaelin C.M., Burgeson R.E., Pardee A.B., Sager R. (1998). Down-Regulation of Laminin-5 in Breast Carcinoma Cells. Mol. Med..

[6] Lin T.C., Yang C.H., Cheng L.H., Chang W.T., Lin Y.R., Cheng H.C. (2019). Fibronectin in Cancer: Friend or Foe. Cells.

[7] Chintala S.K., Sawaya R., Gokaslan Z.L., Fuller G., Rao J.S. (1996). Immimohistochemical Localization of Extracellular Matrix Proteins in Human Glioma, Both in Vivo and in Vitro. Cancer Lett..

[8] Higuchi M., Ohnishi T., Arita N., Hiraga S., Iwasaki H., Mori S., Hayakawa T. (1991). Immunohistochemical Localization of Fibronectin, Laminin and Fibronectin-Receptor in Human Malignant Gliomas. In Relation to Tumour Invasion. Brain Nerve.

[9] Yu Q., Xue Y., Liu J., Xi Z., Li Z., Liu Y. (2018). Fibronectin Promotes the Malignancy of Glioma Stem-like Cells via Modulation of Cell Adhesion, Differentiation, Proliferation and Chemoresistance. Front. Mol. Neurosci..

[10] Wang J., Yan S., Chen X., Wang A., Han Z., Liu B., Shen H. (2022). Identification of Prognostic Biomarkers for Glioblastoma Based on Transcriptome and Proteome Association Analysis. Technol. Cancer Res. Treat..

[11] Payne L.S., Huang P.H. (2013). The Pathobiology of Collagens in Glioma. Mol. Cancer Res..

[12] Noreen R., Chien C.C., Chen H.H., Bobroff V., Moenner M., Javerzat S., Hwu Y., Petibois C. (2013). FTIR Spectro-Imaging of Collagen Scaffold Formation during Glioma Tumour Development. Anal. Bioanal. Chem..

[13] Bellail A.C., Hunter S.B., Brat D.J., Tan C., van Meir E.G. (2004). Microregional Extracellular Matrix Heterogeneity in Brain Modulates Glioma Cell Invasion. Int. J. Biochem. Cell B.

[14] Tso C.L., Shintaku P., Chen J., Liu Q., Liu J., Chen Z., Yoshimoto K., Mischel P.S., Cloughesy T.F., Liau L.M. (2006). Primary Glioblastomas Express Mesenchymal Stem-like Properties. Mol. Cancer Res..

[15] Samuel M.S., Lopez J.I., McGhee E.J., Croft D.R., Strachan D., Timpson P., Munro J., Schröder E., Zhou J., Brunton V.G. (2011). Actomyosin-Mediated Cellular Tension Drives Increased Tissue Stiffness and β-Catenin Activation to Induce Epidermal Hyperplasia and Tumour Growth. Cancer Cell.

[16] Godard S., Getz G., Delorenzi M., Farmer P., Kobayashi H., Desbaillets I., Nozaki M., Diserens A.C., Hamou M.F., Dietrich P.Y. (2003). Classification of Human Astrocytic Gliomas on the Basis of Gene Expression: A Correlated Group of Genes with Angiogenic Activity Emerges As a Strong Predictor of Subtypes. Cancer Res..

[17] Falkowski P., Mrozek P., Lukaszewski Z., Oldak L., Gorodkiewicz E. (2021). An Immunosensor for the Determination of Cathepsin s in Blood Plasma by Array Spri—A Comparison of Analytical Properties of Silver–Gold and Pure Gold Chips. Biosensors.

[18] Sankiewicz A., Romanowicz L., Laudanski P., Zelazowska-Rutkowska B., Puzan B., Cylwik B., Gorodkiewicz E. (2016). SPR Imaging Biosensor for Determination of Laminin-5 as a Potential Cancer Marker in Biological Material. Anal. Bioanal. Chem..

[19] Qu J.H., Ordutowski H., van Tricht C., Verbruggen R., Barcenas Gallardo A., Bulcaen M., Ciwinska M., Gutierrez Cisneros C., Devriese C., Guluzade S. (2022). Point-of-Care Therapeutic Drug Monitoring of Adalimumab by Integrating a FO-SPR Biosensor in a Self-Powered Microfluidic Cartridge. Biosens. Bioelectron..

[20] Singh G.P., Nigam R., Tomar G.S., Monisha M., Bhoi S.K., Arulselvi S., Sengar K., Akula D., Panta P., Anindya R. (2018). Early and Rapid Detection of UCHL1 in the Serum of Brain-Trauma Patients: A Novel Gold Nanoparticle-Based Method for Diagnosing the Severity of Brain Injury. Analyst.

[21] Tian Y., Chen Y., Song D., Liu X., Bi S., Zhou X., Cao Y., Zhang H. (2005). Acousto-Optic Tunable Filter-Surface Plasmon Resonance Immunosensor for Fibronectin. Anal. Chim. Acta.

[22] Dutra R.F., Kubota L.T. (2007). An SPR Immunosensor for Human Cardiac Troponin T Using Specific Binding Avidin to Biotin at Carboxymethyldextran-Modified Gold Chip. Clin. Chim. Acta.

[23] Jung S.H., Kong D.H., Park J.H., Lee S.T., Hyun J., Kim Y.M., Ha K.S. (2010). Rapid Analysis of Matrix Metalloproteinase-3 Activity by Gelatin Arrays Using a Spectral Surface Plasmon Resonance Biosensor. Analyst.

[24] Shoji A., Kabeya M., Sugawara M. (2011). Real-Time Monitoring of Matrix Metalloproteinase-9 Collagenolytic Activity with a Surface Plasmon Resonance Biosensor. Anal. Biochem..

[25] Lin S., Shih-Yuan Lee A., Lin C.-C., Lee C.-K. (2007). Determination of Binding Constant and Stoichiometry for Antibody-Antigen Interaction with Surface Plasmon Resonance. Curr. Proteom..

[26] Sankiewicz A., Romanowicz L., Pyc M., Hermanowicz A., Gorodkiewicz E. (2018). SPR Imaging Biosensor for the Quantitation of Fibronectin Concentration in Blood Samples. J. Pharm. Biomed. Anal..

[27] Sankiewicz A., Lukaszewski Z., Trojanowska K., Gorodkiewicz E. (2016). Determination of Collagen Type IV by Surface Plasmon Resonance Imaging Using a Specific Biosensor. Anal. Biochem..

[28] Hlubina P., Ciprian D. (2017). Spectral Phase Shift of Surface Plasmon Resonance in the Kretschmann Configuration: Theory and Experiment. Plasmonics.

[29] Baeten K.M., Akassoglou K. (2011). Extracellular Matrix and Matrix Receptors in Blood–Brain Barrier Formation and Stroke. Dev. Neurobiol..

[30] Scanlon C.S., van Tubergen E.A., Inglehart R.C., D’Silva N.J. (2013). Biomarkers of Epithelial-Mesenchymal Transition in Squamous Cell Carcinoma. J. Dent. Res..

[31] Hindermann W., Berndt A., Haas K.M., Wunderlich H., Katenkamp D., Kosmehl H. (2003). Immunohistochemical Demonstration of the Γ2 Chain of Laminin-5 in Urinary Bladder Urothelial Carcinoma Impact for Diagnosis and Prognosis. Cancer Detect. Prev..

[32] Kiyoshima K., Oda Y., Kinukawa N., Naito S., Tsuneyoshi M. (2005). Overexpression of Laminin-5 Γ2 Chain and Its Prognostic Significance in Urothelial Carcinoma of Urinary Bladder: Association with Expression of Cyclooxygenase 2, Epidermal Growth Factor, and Human Epidermal Growth Factor 2. Hum. Pathol..

[33] Farha K.M.M.A., Janknegt R.A., Kester A.D.M., Arends J.W. (1993). Value of Immunohistochemical Laminin Staining in Transitional Cell Carcinoma of Human Bladder. Urol. Int..

[34] Schapers R.F., Pauwels R.P., Havenith M.G., Smeets A.W., van den Brandt P.A., Bosman F.T. (1990). Prognostic Significance of Type IV Collagen and Laminin Immunoreactivity in Urothelial Carcinomas of the Bladder. Cancer.

[35] Daher N., Abourachid H., Bove N., Petit J., Burtin P. (1987). Collagen IV Staining Pattern in Bladder Carcinomas: Relationship to Prognosis. Br. J. Cancer.

[36] Brunner A., Tzankov A. (2007). The Role of Structural Extracellular Matrix Proteins in Urothelial Bladder Cancer (Review). Biomark. Insights.

[37] Menéndez V., Fernández-Suárez A., Galán J.A., Pérez M., García-López F. (2005). Diagnosis of Bladder Cancer by Analysis of Urinary Fibronectin. Urology.

[38] Ioachim E., Michael M., Stavropoulos N.E., Kitsiou E., Salmas M., Malamou-Mitsi V. (2005). A Clinicopathological Study of the Expression of Extracellular Matrix Components in Urothelial Carcinoma. BJU Int..

[39] Malmstrom P.U., Larsson A., Johansson S. (1993). Urinary Fibronectin in Diagnosis and Follow-up of Patients with Urinary Bladder Cancer. Br. J. Urol..

[40] Hegele A., Heidenreich A., Varga Z., von Knobloch R., Olbert P., Kropf J., Hofmann R. (2003). Cellular Fibronectin in Patients with Transitional Cell Carcinoma of the Bladder. Urol. Res..

